# Tunable single-photon frequency conversion in a Sagnac interferometer

**DOI:** 10.1038/srep03555

**Published:** 2013-12-19

**Authors:** Wei-Bin Yan, Jin-Feng Huang, Heng Fan

**Affiliations:** 1 Beijing National Laboratory for Condensed Matter Physics, Institute of Physics, Chinese Academy of Sciences, Beijing 100190, China; 2 State Key Laboratory of Theoretical Physics, Institute of Theoretical Physics, Chinese Academy of Sciences, Beijing 100190, China; 3 Department of Physics and Institute of Theoretical Physics, The Chinese University of Hong Kong, Shatin, Hong Kong Special Administrative Region, People's Republic of China

## Abstract

Quantum information carriers like photons might be manipulated, stored and transmitted in different quantum systems. It is important to integrate those systems efficiently. The capability of converting photons from one wavelength to another wavelength is a key requirement for combining the photons in telecommunications band for quantum transmission and the photons in near-visible band for quantum storage. Here, we investigate the tunable single-photon frequency conversion in the five-level emitter-Sagnac interferometer system. We show that the efficient single-photon conversion can be achieved in this scheme, at the same time, the frequencies of the input and output photons can be tuned in a large scale by controlling the frequencies and Rabi frequencies of the external driving fields. The realization of this scheme may lead to the efficient combination of quantum storage system with the quantum communication system.

Quantum frequency conversion^1^,^2^ is a nonlinear process transducing an input beam of light with a given frequency into an output beam of light with a different frequency. It has many critical applications in quantum communication and quantum information processing^3^,^4^,^5^,^6^,^7^,^8^,^9^,^10^,^11^,^12^,^13^,^14^,^15^,^16^,^17^,^18^,^19^. The highly efficient photon frequency conversion can be achieved in the large-flux limit^20^,^21^. Recently, the authors in Ref. 22,23 pointed out that the highly efficient photon frequency conversion at low light levels has not been achieved. To achieve this conversion, they proposed a realizable scheme that a Sagnac interferometer^24^,^25^,^26^,^27^ coupled to a three-level quantum emitter. They show that the highly frequency conversion at single photon level can be achieved due to the interference when the coupling strengths between the different atomic transitions to the waveguide loop of the Sagnac interferometer are equal, and additionally with resonant condition which has fixed the photon frequencies. We notice that in the highly efficient photon frequency conversion, both at the large-flux limit or low-light level, the frequencies of the input and output photons are limited in a very small scale. Yet, the tunable highly efficient frequency conversion in which both the frequencies of the input and output photons can be tuned in a large scale has not been explored.

Here we propose a scheme to achieve the tunable efficient single-photon frequency conversion. We show that both of the frequencies of the input and output photons can be tuned in a large scale by adjusting the system parameters in the efficient single-photon frequency conversion. When the frequency of the output photon is tuned higher than the input photon, the up conversion is achieved, while the down conversion can be achieved in the opposite situation. We demonstrate this control with a five-level emitter coupled to a Sagnac Interferometer. Compared to the Sagnac interferometer coupled to a three-level emitter^22^,^23^, we show that the efficient frequency conversion can be achieved in either resonance or off-resonance case. In the resonance case, the condition that the different atomic transition-waveguide loop coupling strengths are equal is not necessarily essential to obtain a high conversion efficiency. This is more realizable under practical conditions. In particular, the efficient conversion can also be achieved for off-resonance case which permits tunable photon frequencies.

The structure of the system under consideration is shown in Fig. 1. The Sagnac interferometer consists of a 50:50 coupler and a waveguide loop. It creates a superposition of two counter-propagating photon states when a single photon is injected into the setup. To avoid the output photon returning to the light source, a supplementary route which is not illustrated here is necessary, as shown in^22^,^23^. The emitter which will be mentioned as an atom below can be a real atom or a manual atom-like object. The two atomic long-live states are denoted by |*b*〉 and |*c*〉, and the excited states |*a*〉, |*d*〉, and |*f*〉. The atomic level frequencies are represented by *ω_i_*(*i* = *a, b, c, d, f*). The atomic transitions |*a*〉 ↔ |*b*〉 and |*d*〉 ↔ |*c*〉 are coupled to the photons in the waveguide loop with strengths *g*_1_ and *g*_2_, respectively. The coupling strengths are assumed to be independent of the waveguide wave number which is equivalent to the Weisskopf-Wigner approximation. We employ two external classical fields with frequencies (Rabi frequencies) 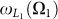 and 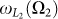 to drive the atomic transitions |*a*〉 ↔ |*f*〉 and |*d*〉 ↔ |*f*〉, respectively. The five-level atomic configurations have been been studied extensively, for example^28^,^29^. In this report, we first derive the transport property of the five-level atom coupled to the waveguide loop. We then find the system output state of the atom coupled to the whole Sagnac interferometer by the scattering matrix to study the controllable single-photon frequency conversion. The scattering matrix of the Sagnac interferometer is *S* = *S_c_S_l_S_c_*, with 
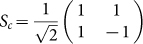
 representing the beam splitter, and 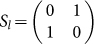 representing that the photon goes out from another port different from the previous input port after a round in the waveguide loop. The waveguide loop can be treated as a one dimensional waveguide which has been studied extensively, for example, see^30^,^31^,^32^,^33^,^34^,^35^,^36^,^37^,^38^,^39^,^40^,^41^,^42^,^43^,^44^,^45^,^46^,^47^,^48^,^49^,^50^.

The time-independent Hamiltonian of the atom coupled to a waveguide reads, 
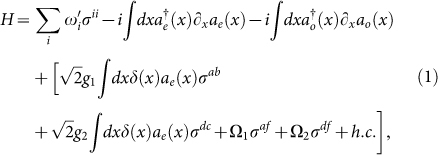
with 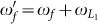, 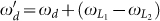, 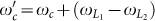 and *σ^ij^* = |*i*〉 〈*j*| denoting the atomic raising, lowering and energy level population operators. Here we have taken 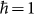, and the photonic group velocity *v_g_* = 1. It can be seen that the external fields shift the atomic levels. The expressions of the even and odd operators are 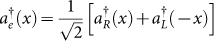 and 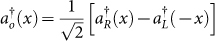, with the operator 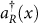 and 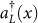 creating a clockwise and counterclockwise moving photon in the waveguide^32^,^33^, respectively. Note that the effective atomic frequency, 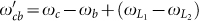, is related to the external field frequencies. We assume that, initially, the atom is in the state |*b*〉, and a photon with the wave number *k* is injected into the waveguide loop. After scattering, the atom is in the state |*b*〉 or |*c*〉, with the corresponding wave number of the output photon *k* and *k*′, respectively. The former corresponds to the elastic scattering and the latter to the inelastic scattering. For the inelastic scattering, the frequency of the output photon depends on the external field frequencies. Therefore, it is essential to make sure that the input photon is merely inelastically scattered for various values of the external field frequencies to achieve the tunable frequency conversion.

## Results

### Single-photon frequency conversion properties

For an input photon split by the 50:50 coupler, the superposition of the clockwise and counterclockwise moving states can be prepared in the waveguide loop. In certain cases, the interference resulting from the superposition has a constructive effect on the inelastic scattering and a destructive effect on the elastic scattering. Once the relative phase between the photonic clockwise and counterclockwise moving states is zero, the scattered state can be obtained as 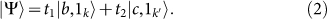
with 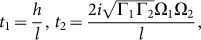

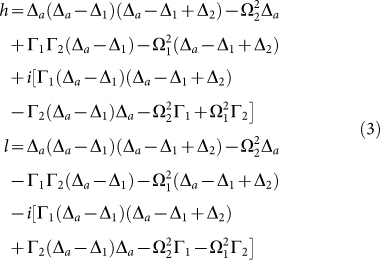
where Δ*_a_* = *ω_a_* − *k*, 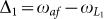, 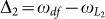, and 
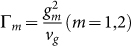
 representing the atomic decay rate into the waveguide loop due to the coupling. When *t*_2_ = 1, the inelastic scattering process converts the input single photon into an output photon of the wave number *k*′ with unity conversion efficiency.

**Figure 2:**
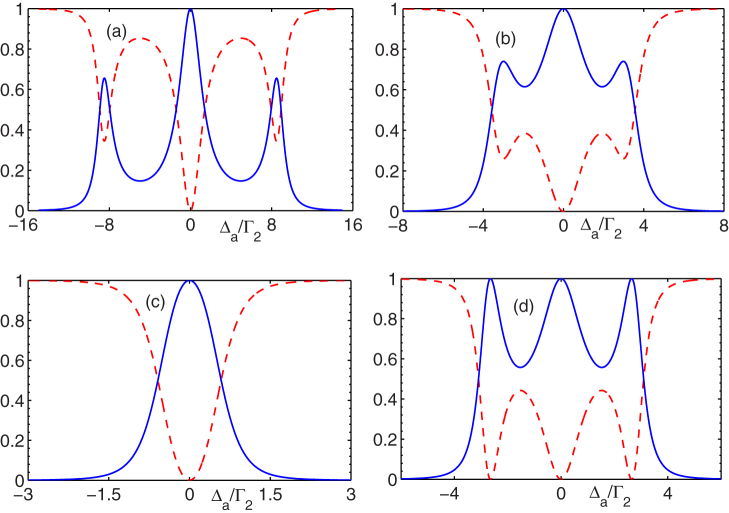
Frequency efficiency conversion properties against the input-photon frequency. The red dashed lines are |*t*_1_|^2^ and the blue solid lines are |*t*_2_|^2^. We have taken 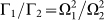 and Δ_1_ = Δ_2_ = 0 in all of the plots. The respective parameters are (a)Γ_1_ = 2Γ_2_, 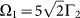, (b)Γ_1_ = 2Γ_2_, 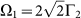, (c)Γ_1_ = 2Γ_2_, 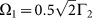, (d)Γ_1_ = Γ_2_, Ω_1_ = 2Γ_2_.

The control of the frequency of the output photon for a high conversion efficiency is our prime concern. The frequency of the output photon after the inelastic scattering is obtained as 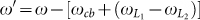 which can be controlled by tuning the frequencies of the external lasers. This can be understood by the energy conservation. When 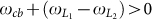, the down conversion can be achieved after the inelastic scattering, and when 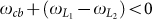, the up conversion can be achieved. Obviously, if the resonance condition is satisfied i.e., Δ*_a_* = Δ_1_ = Δ_2_ = 0, we can obtain the unity conversion efficiency when 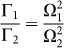. The coupling strength *g*_1_ is usually different from the other strength *g*_2_ because they depend on the atomic dipole. Hence, the controllable Rabi frequencies enable us to obtain a unity conversion efficiency in the resonance case. Fig. 2 shows the conversion properties |*t*_1_|^2^ and |*t*_2_|^2^ against the frequency of the input single photon when the external lasers drive the atomic transitions resonantly when 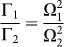. For small Rabi frequencies, the spectra are shaped like the Lorentzian line. The spectra split with the increasing Rabi frequencies. When Γ_1_ = Γ_2_ = Γ, and 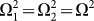, we can find 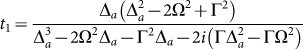. Obviously, when Γ^2^ − 2Ω^2^ ≥ 0, the unity conversion efficiency can be achieved only when the input photon interacts with the atom resonantly. However, when Γ^2^ − 2Ω^2^ < 0, the unity conversion efficiency can also be obtained even when the input photon is off-resonant to the atomic transition as shown in Fig. 2(d).

### Tunable single-photon frequency conversion

**Figure 3:**
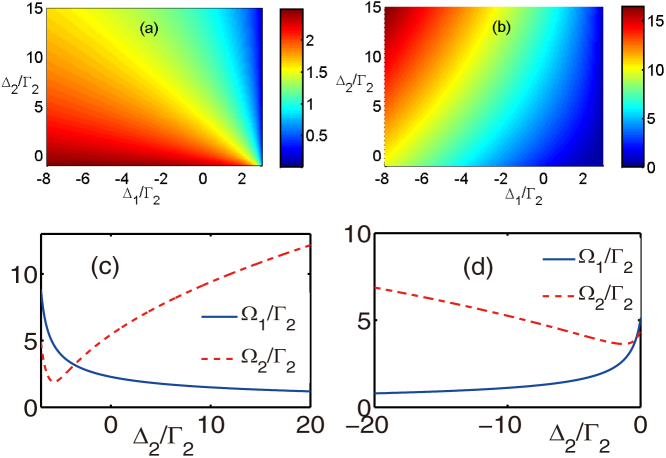
The values of Rabi frequencies against the frequencies of external driving lasers when the photon conversion efficiency is unity. (a) and (b) are Ω_1_ and Ω_2_ against the two laser frequencies, respectively. The parameters are Δ*_a_* = 3Γ_2_, Γ_1_ = 2Γ_2_. We take Δ_1_ = −3Γ_2_ in (c), and Δ_1_ = 5Γ_2_ in (d).

In the case discussed above, the external classical frequencies are fixed and then can not be tuned to satisfy the resonance condition. In order to achieve the tunable frequency of the converted output photon, the unity conversion efficiency in the off-resonance case is required. In the detuned case, the condition *t*_1_ = 0 requires 
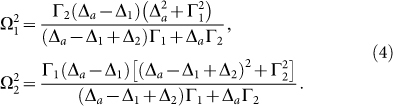
Therefore, the conditions 
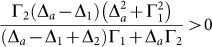
 and 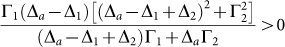 are essential to obtain a unity conversion efficiency. Although these conditions can not be satisfied for any arbitrary value of the frequencies of the external fields, they can be fulfilled in a large range of the frequency values. This feasible range is enough for the adjusting of the converted-photon frequency in a wide scale. To explain this, we plot the Rabi frequencies Ω_1_ and Ω_2_ against the frequencies of the external fields when *t*_1_ = 0 in Fig. 3. In Fig. 3(a) and 3(b), we show the required Rabi frequencies when we adjust both the external frequencies together. Fig. 3(c) and 3(d) show the Rabi frequency requirement when we adjust one of the external frequency while the other frequency is fixed. Fig. 3 shows that for the large scale of the external laser frequencies, the essential conditions above can be satisfied and the appropriate values of the Rabi frequencies can be found. Therefore, we can control the frequency of the converted output photon by controlling the frequencies of the external laser and tune the Rabi frequencies to obtain a unity conversion efficiency. Although the injected photon is not resonant with the atom, the suitable parameters of the external lasers can ensure the conversion complete. The frequency conversion process can be understood as a photon trapping process. After the inelastic scattering, the injected photon *a* is trapped and the atom is in the state |*c*〉, with another photon *b* created. Besides, the trapped photon can be retrieved by injecting the photon *b*. It means that, the photon is trapped for a complete conversion. The retrieval processing corresponds to the complete conversion *b* → *a*. The retrieval efficiency can be computed when the atomic initial state is |*c*〉 by a similar calculation done above. Obviously, the retrieval efficiency can be unity under a suitable condition.

### Dissipation case

**Figure 4:**
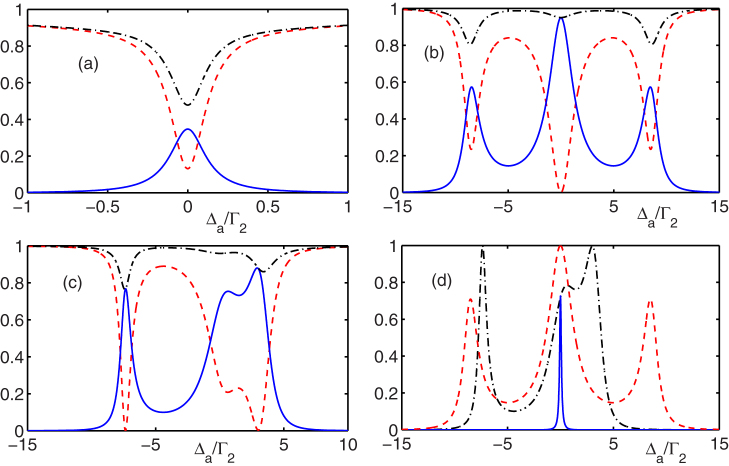
The photon conversion properties influenced by dissipation. (a), (b) and (c) show the probabilities |*t*_1_|^2^, |*t*_2_|^2^, and the total probability |*t*_1_|^2^ + |*t*_2_|^2^, which correspond to the red dashed lines, blue solid lines, and black dashed dotted lines, respectively. (d) shows the probability 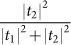. The blue solid line, red dashed line, and black dashed dotted line denote the situation as shown in (a), (b) and (c), respectively. For all the plots, the dissipation rate is taken 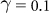 and the coupling strengths Γ_1_ = 2Γ_2_. The respective parameters are (a) 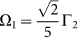, 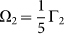, Δ_1_ = Δ_2_ = 0, (b) 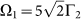, 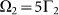, Δ_2_ = 5Γ_2_, Δ_1_ = Δ_2_ = 0, (c) 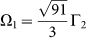, 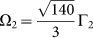, Δ_1_ = Δ_2_ = −4Γ_2_.

The intrinsic dissipation is harmful to achieve the unity conversion efficiency. This dissipation can be incorporated by introducing the nonhermitian Hamiltonian 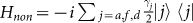 in the quantum jump picture, with 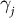 being the decay rate to other modes except the mode of the waveguide loop from the level |*j*〉 for a real atom and being the decay rate plus dephase rate for a manual atom-like object. As shown above, a complete conversion can be achieved in the resonance and off-resonance cases under the ideal condition. Fig. 4(a), 4(b) and 4(c) plot the conversion properties in both the cases after considering the dissipation. The strong coupling and large detuning can tolerate the dissipation better. Fig. 4(d) plots the probability 
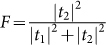
. The high conversion efficiencies can be obtained in the case as shown in Fig. 4(b) and Fig. 4(c). The probability *F* can be nearly unity which means that the input photon is dissipated and converted, and little elastic scattering exists. We note another restricting condition that the Rabi frequencies can not be too small in order to tolerate the dissipation. Fortunately, after considering this condition, the tunable frequency scale of the output photon is little affected, which can be understood from Fig. 3. We have study the case that the input light is monochromatic. For a input pulse with finite bandwidth, the conversion efficiency decreases, which can be seen in Fig. 4(c). To achieve the efficient single-photon frequency conversion, the narrow bandwidth of the input pulse is necessary.

## Discussion

We propose a tunable single-photon frequency conversion scheme with high efficiency. The inelastic scattering shifts the frequency of the input photon. Especially, in the off-resonance case, the frequency shift can be tuned by adjusting the external classical fields. Thus, the output frequency is tunable. The dissipation will in general diminish the conversion efficiency from the unity and the output photon is mostly the inelastically scattered photon. Having considered the dissipation and the narrow bandwidth of the input pulse, the high efficiency can also be achieved. The realization of this scheme may combine the quantum information processing system with the long-distance quantum communication system.

## Methods

The one-excitation state of the waveguide-atom system can be written as 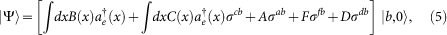
where *B*(*x*), *C*(*x*), *A*, *F*, and *D* are amplitude probabilities, and |*b*, 0〉 represents that the atom is in the state |*b*〉 and the photon number in the waveguide is zero. Under the ansatz *B*(*x*) = [*θ*(−*x*) + *t*_1_*θ*(*x*)]*e^ikx^* and *C*(*x*) = *t*_2_*θ*(*x*)*e^ik′x^*, we can find the solution of the time-independent Shrödinger equation *H*|Ψ〉 = *E*|Ψ〉. The stationary state evolves with time as |Ψ(*t*)〉 = *e*^−*iEt*^|Ψ〉. After calculation^43^, the transport properties *t*_1_ and *t*_2_ are obtained as in Eq. (3).

Going back to the clockwise and counterclockwise picture from the even and odd picture, the scattering matrix of the emitter coupled to the waveguide loop can be derived from *t*_1_ and *t*_2_^32^,^33^ and then the whole system scattering matrix can be calculated. As long as any one of the Rabi frequencies {Ω_1_, Ω_2_} is zero, the frequency conversion efficiency is zero due to the fact that the atomic transition |*d*〉 ↔ |*c*〉 decouples from the photon in the waveguide and hence the inelastic scattering vanishes. In detail, when Ω_1_ = 0, we can find 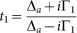 and *t*_2_ = 0, which is the same as a two-level system coupled to the waveguide^30^,^31^,^32^,^33^. And when Ω_2_ = 0, we can find 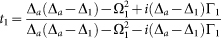 and *t*_2_ = 0, corresponding to a Λ three-level atom coupled to the waveguide^44^. This also reveals that the frequency conversion can be switched off by shutting off the external classical field, which is equivalent to the control of the relative phase shift between the clockwise and counterclockwise moving photon. When the relative phase is *π*, an odd-mode quasi particle is prepared in the waveguide loop and the destructive interference makes the frequency conversion efficiency zero.

The relationship |*t*_1_|^2^ + |*t*_2_|^2^ = 1 can be easily checked. The maximal frequency conversion efficiency is 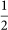 when a photon moves only clockwise or only counterclockwise towards the atom in the waveguide loop. In this case, the output state has the form of 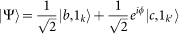 with *ϕ* being a real number, which is a maximally entangled state.

## Figures and Tables

**Figure 1:**
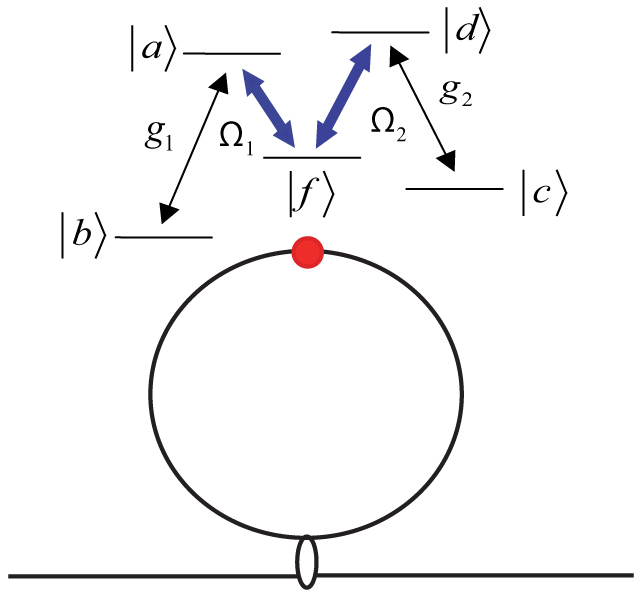
A Sagnac interferometer coupled to a five-level emitter. Two external classical fields are employed to drive the atomic transitions.

## Notes

**Correspondence** Correspondence and requests for materials should be addressed to H.F. or W.-B. Y.
